# Obstetric care providers assessing psychosocial risk factors during pregnancy: validation of a short screening tool – the KINDEX Spanish Version

**DOI:** 10.1186/s13034-014-0030-7

**Published:** 2014-12-23

**Authors:** Andria Spyridou, Maggie Schauer, Martina Ruf-Leuschner

**Affiliations:** University of Konstanz, Konstanz, Germany; Vivo International (www.vivo.org), Konstanz, Germany

**Keywords:** Prenatal assessment, Psychosocial risks, KINDEX Spanish, Pregnancy, Early attention

## Abstract

**Background:**

High levels of stress due to diverse psychosocial factors have a direct impact on the mothers’ wellbeing during pregnancy and both direct and indirect effects on the fetus. In most cases, psychosocial risk factors present during pregnancy will not disappear after delivery and might influence the parent-child relationship, affecting the healthy development of the offspring in the long term.

We introduce a short innovative prenatal assessment to detect psychosocial risk factors through an easy to use instrument for obstetrical medical staff in the daily clinical practice, the KINDEX Spanish Version.

**Methods:**

In the present study midwives and gynecologists interviewed one hundred nineteen pregnant women in a public health center using the KINDEX Spanish Version. Sixty-seven women were then randomly selected to participate in an extended standardized validation interview conducted by a clinical psychologist using established questionnaires to assesses current stress (ESI, PSS-14), symptoms of psychopathology (HSCL-25, PDS) and traumatic experiences (PDS, CFV). Ethical approval was granted and informed consent was required for participation in this study.

**Results:**

The KINDEX sum score, as assessed by medical staff, correlated significantly with stress, psychopathology and trauma as measured during the clinical expert interview. The KINDEX shows strong concurrent validity. Its use by medical staff in daily clinical practice is feasible for public health contexts. Certain items in the KINDEX are related to the respective scales assessing the same risks (e.g.PSS-4 as the shorter version of the PSS-14 and items from the ESI) used in the validation interview.

**Conclusions:**

The KINDEX Spanish Version is a valid tool in the hands of medical staff to identify women with multiple psychosocial risk factors in public health settings. The KINDEX Spanish Version could serve as a base-instrument for the referral of at-risk women to appropriate psychosocial intervention. Such early interventions could prove pivotal in preventing undesirable mother-child relationships and adverse child development.

## Background

A lifetime of healthy brain development starts long before birth, during pregnancy [1-3]. Moreover, there is ample evidence supporting the impact of different psychosocial risk factors on the unborn child [4,5] and the newborns later brain development [6]. Nevertheless, the transfer of this research knowledge into practice only began in the past decade [7,8]. Worldwide, there are only a few studies reporting the development, evaluation and implementation of screening tools for psychosocial risk factors in pregnant women and subsequent intervention and prevention programs in community health centers in the U.S. [9], Australia [10] and Canada [11].

Today, several risk factors have been identified as crucial for both maternal, fetal and later child development. Adverse neonatal and obstetric outcomes have been linked with *maternal stress* [12,13], *mental health problems* of the mother [14-16], and *intimate partner violence (IPV)* [17]. Depression is the strongest predictor of poor psychological well-being in pregnant women [18] and of lower quality of maternal-fetal attachment [19]. In contrast, *positively attached* mothers have better prenatal health practices, such as abstinence from smoking, alcohol and drug abuse [20]. The severe impact of *alcohol, tobacco and drug consumption* during pregnancy is well-known [21,22]. Several factors have been associated with elevated alcohol and drug consumption such as deteriorated mental health, physical health, peer and family relations, and educational status among others [23].

The presence of psychosocial risks produce higher perceived stress in women from *low socioeconomic status* [24], *adolescent or very young mothers (<20 years of age)* [25], in *immigrant* [26] and *refugees* from war-torn societies that often are diagnosed with PTSD [27,28]. These social groups often lack social support [29], a stress mediating factor [30] present higher levels of IPV, drug abuse [31] child maltreatment and present worse parenting skills [32]; all the above conditions result in poorer birth outcomes [33-35].

Child neurodevelopment [36] and child behavioral problems linked to altered HPA activity [37] have been related to stress and maternal mood [38,39]. Recent studies have also revealed long-term biological effects of IPV exposure during pregnancy; the methylation status of the GR gene in adolescent children is influenced by maternal experience of IPV during pregnancy [5]. Offspring’s poor behavioral trajectories and elevated physical abuse, separation from parents and changes in family composition have been reported in children of mothers that have *experienced violence during childhood* [40,41], a frequently undisclosed risk for the etiology of depressive and posttraumatic stress symptoms in pregnant women [42]. Mediated pathways have been found between maternal childhood abuse (MCA) to substance abuse and offspring victimization, perpetrating the vicious cycle of violence [43,44]. MCA also predict increase in offspring’s externalizing behavior, suggesting an impact in subsequent generations [45].

Early identification and appropriate intervention may work to ameliorate the adverse effects of such psychosocial risks [46,47]. Nevertheless, very little research focuses on the development and evaluation of screening tools for psychosocial risk factors during pregnancy. As a consequence, up to 50% of depression cases will go unnoticed [48] and only 18% of women diagnosed with depression will seek professional help [49].

The multiple risks that may be present during pregnancy demand the development and use of multidimensional assessment tools. Johnson et al (2012) in a review of the existing tools for factors influencing perinatal mental health assessment revealed 6 valid instruments. This review assessed the reliability, validity, sensibility and specificity and normative data when these were reported by the authors. The results revealed that tools where assessing factors from 3 domains [Contextual Assessment of Maternity Experience (CAME), to 26 [Camberwell Assessment of Need—Mothers (CAN-M)]. All the assessment tools were ‘*not recommended*’ due to the existence of ‘unacceptable’ reliability, validity or normative data based on the Hammil scoring system.

In Canada, a multidisciplinary team of health professionals developed an evidenced-based prenatal risk assessment program in order to identify and manage women and families in psychosocial risk using the Antenatal Psychosocial Health Assessment (ALPHA-Form) that uses 35 items to detect 15 risk factors for postnatal adverse psychosocial outcomes [50].

In a randomized control trial in four communities in Ontario, Canada, midwives, obstetricians and family physicians using the ALPHA form in place of traditional care procedures were more likely to detect risks in women that related to postpartum outcomes than health providers in the control group [50]. Another study that applied the ALPHA-Form, this time in Australia, both expectant women and midwives had a positive reception of the program and the identification of high-risk women was much more efficient than traditional assessments in obstetric care [51]. Despite its demonstrated feasibility in different cultural contexts, the specificity, sensitivity, positive and negative predictive values of the ALPHA Form have not yet been assessed [52].

The Antenatal Risk Questionnaire (ANRQ) was both developed through consultations with midwives and health professionals working in a maternity hospital and by Austin et al. (2013) [10]. Johnson et al., (2012) found this tool to fulfill more of the requirements than any of the others assessed [52]. The ANRQ is composed of 12 items retrieved from the original 23 Pregnancy Risk Questionnaire (PRQ) [53] and assesses seven psychosocial risk domains: emotional support from subject’s own mother in childhood, past history of depressed mood or mental illness and treatment received, perceived level of support available after birth of the baby, partner emotional support, life stresses in the past 12 months, personality (anxious or perfectionistic traits) and history of abuse (emotional, physical and sexual). It has a possible rating score from a minimum of 5 to a possible maximum of 62 and the authors suggest a clinically relevant cutoff of 23. The psychometric properties of the tool include acceptable sensitivity (0.62) and specificity (0.64), it has high face and construct validity of the factors assessed, and has high acceptability amongst midwives and pregnant women, nevertheless it has low positive and negative predictive values emotional support from subject’s own mother in childhood, past history of depressed mood or mental illness and treatment received, perceived level of support available after birth of the baby, partner emotional support, life stresses in the past 12 months, personality (anxious or perfectionistic traits) and history of abuse (emotional, physical and sexual). It has a possible rating score from a minimum of 5 to a possible maximum of 62 and the authors suggest a clinically relevant cutoff of 23. The psychometric properties of the tool include acceptable sensitivity (0.62) and specificity (0.64), it has high face and construct validity of the factors assessed, and has high acceptability amongst midwives and pregnant women, nevertheless it has low positive and negative predictive values [52]. The ANRQ conjunctly with the symptom-based Edinburgh Depression Scale has been used within the psychosocial risk assessment model (PRAM) embedded in the integrated perinatal care context at the Royal Hospital for Women in Sydney, Australia on 2,142 women. Based on this assessment, the researchers computed a Psychosocial Risk Index (PRI) in order to provide individualized care planning [7]. The follow-up study at 2 or 4 months postpartum revealed a positive predictive value for postnatal development of depression of 0.3, rather low. Authors conclude that the instrument could be used with a symptom-based instrument such as Edinburgh Postnatal Depression Scale or routine questions concerning drug and alcohol use and domestic violence to provide a “routine screening intervention” [10].

In spite of substantial research on the development and evaluation of prenatal psychosocial risk factors, the literature is not without its limitations. The need for longitudinal research examining the predictive validity of the tools for child development is outstanding. The severe effects the presence of psychosocial risks present during the perinatal period to maternal mental health and infant development have been replicated many times. These findings point to the emergent need for the development of easy to apply and efficient tools in order to boost prevention of negative outcomes in maternal-infant/child populations.

In this study we evaluate the Spanish Version of the KINDEX. Originally the KINDEX was developed in German after a critical review of evidence-based literature on psychosocial risk factors during pregnancy that have an adverse effect on both the maternal mental health and child development later on. Historically, assessment tools have been focused on the presence of risks that could screen or predict maternal mental health and influence the infant [52], in change the KINDEX was developed by a panel of experts through a comprehensive literature review on risk factors for the maternal mental health and child development in the long run. The tool assesses 11 risk areas during pregnancy and is designed to be used by medical staff in their everyday clinical practice. Similarly to the ANRQ, it assess presence of psychological factors and the experience of adversities in the past such as mother’s sexual and physical abuse, but, it additionally assess the maternal and paternal fetal attachment and takes into account social risks, such as financial difficulties, immigrant/refugee origin of the parents, maternal age and medical risks. Cross-sectional and longitudinal validation studies in Germany showed good psychometric properties, high prospective validity and a good implementation feasibility Schauer, M., Ruf-Leuschner M.: KINDEX: Prenatal assessment of psychosocial risk factors for development – the Konstanz INDEX, submitted.

The aim of our study was two-fold. First, we want to explore whether the use of the KINDEX is feasible in the daily practice of medical staff providing prenatal care in Spain in a representative sample of the general population.

Second, we wanted to examine the criterion-related concurrent validity of the KINDEX by assessing the relation of the KINDEX interview with the validation interview carried out by an expert clinical psychologist.

The final objective was to achieve the cultural adaptation of the KINDEX Spanish Version and to offer a valid tool for the psychosocial risk assessment to the obstetric care providers.

## Methods

### Translation and adaptation procedure of the KINDEX

The translation procedure of the KINDEX was based on the World Health Organization guidelines for translation process and adaptation of instruments [54]. This was achieved through the following steps: 1) Forward translation by two bilingual health professionals familiar with both the German and Spanish cultures, 2) A panel of four experts, comprised of two bilingual psychologists, one health expert and one translation/adaptation expert, agreed on the adequacy of the translated version. 3) Back translation by two independent bilingual translators with emphasis on the conceptual and cultural equivalence. Only minor discrepancies were found and agreement by the expert’s panel was achieved after small changes. 4) Focus groups with the four medical staff members that collaborated in the study and used the KINDEX in the Maternity Hospital. The Medical staff and translators came to an agreement after discussions on the KINDEX items adequacy.

### Time and place of the study

All interviews conducted by midwives and gynecologists using the KINDEX were carried out between October 2010 and March 2011. KINDEX interviews took place in the different units of the University Hospital Virgen de las Nieves, Maternity Clinic of Granada, Spain. Sixty-two (52.1%) participants were interviewed in the outpatient consultation of the hospital, during their regular doctor’s appointment, forty (33.6%) in the fetal medicine unit, thirteen (10.9%) while they were hospitalized due to high-risk pregnancy and four (3.4%) in the emergency room. Using the Kruskal –Wallis test, no significant difference was found in the KINDEX sum score between participants who were interviewed in different hospital units [*H*(3) = 2.85; *p* = .41]. Validation interviews were carried out by a clinical psychologist between October 2010 and March 2011 in the same Maternity Clinic in a private room provided for the needs of the interview.

### Interviewers

KINDEX: Eight midwives and three gynecologists took part in the study. The midwives interviewed seventy-three (61.3%) pregnant women while forty-six (38.7%) women were interviewed by the gynecologists. No significant difference was found between participants interviewed by gynecologists (M = 4.28; SD = 2.74) and by midwives (M = 4.16 SD = 2.50) with regard to the KINDEX sum score [*t*(117) = .34; *p* = .39].

Validation: All validation interviews were carried out by a PhD-student and clinical psychologist of the Department of Clinical Psychology of the University of Konstanz. The interviewer was blind regarding the KINDEX assessment before the validation interview to avoid any bias. The PhD-student was fluent in Spanish and trained in all standardized instruments at the Center of Excellence for Psychotraumatology at the University of Konstanz, Germany.

### Procedure

To avoid selection bias by gynecologists and midwives, a set of randomization strategies^a^ was applied, when, due to time constraints, it was not possible for the medical staff to ask all pregnant women to participate in the KINDEX interview. Participation requirements included being between 24th and 36th week of gestation and having good comprehensive skills of the Spanish language. Interviewers had to use the KINDEX to interview the participants and not to administrate it as a self-report questionnaire to the pregnant women. Prior to the interview the gynecologist or midwife informed the pregnant woman about the aim of the study, confidentiality and its voluntary nature. Afterwards, the participant was asked to read the information sheet and give her written informed consent to be able to proceed with the interview. All KINDEX interviews took place in privacy without the presence of other family members or the partner. Throughout the entire interview procedure a clinical psychologist of the Department of Clinical Psychology of the University of Konstanz was reachable and had weekly meetings with the group of medical staff collaborating in the study in order to discuss the screening process and clarify any questions and doubts that occurred during the KINDEX interviewing procedure. No emergency occurred due to the interview. The medical staff received a symbolic stipend of 10 Euros to compensate for the time inverted in the study. Patient participants were not compensated for their participation in the KINDEX interview.

A randomized sample of sixty-seven participants was selected to participate in the validation interview by an experienced clinical psychologist. The time gap between the KINDEX and the Validation interview was on average 2.85 weeks (*SD* =1.57, range = 1–7 weeks).

The study received ethical clearance from the Public Foundation of Andalusia for Biomedical Research (FIBAO) and the Ethics Committee of the University Hospital Virgen de las Nieves in Granada.

### KINDEX

The KINDEX was developed at the University of Konstanz, Germany in 2009 [55] based on the current literature on risk factors for healthy child development. Thirty-one items, some with sub-items, which assess 11 different risk factors, compose the KINDEX. It is designed as a short interview (20–30 min) that can be conducted by midwives and gynecologists without any specific training in the psychosocial concepts.

The first risk factor found in the KINDEX is the *mother’s age*, which uses an ordinal scale. Using the age range we created a binary index. When the mother is 21 years or younger it is considered a risk factor. *Migration* is another risk factor that we measure through two binary items (mother’s and father’s country of birth / if not Spain, define). The factor *“single parent”* for the mother is also recoded dichotomously. Financial worries and housing situation items compose the *financial problems* factor. The item referred to “fears concerning financial difficulties” is binary. In addition we asked for the number of rooms and the number of persons living at home. Afterwards we computed a housing index; when less than 0.5 (rooms / persons) is regarded to be a risk factor. *Prenatal bonding* is assessed through 5 items. One binary item was included regarding the planning of the pregnancy. In addition, the mother and father’s joy and worries about the future with their baby is recorded on a 0 (very low) to 10 (very high) scale. The two items of joy and worries are recoded into a binary scale; the upper (worries; 7–10) and lower (joy; 0–3) quartiles are considered to be negative prenatal bonding. *Physical symptoms, complications during pregnancy* and *medical risk factors* are assessed through three binary questions. *Perceived current stress* as experienced by the pregnant woman is measured through an ordinal scale, the PSS-4 (Perceived Stress Scale) [56]. The PSS-4 is a standardized instrument that collects, through a four-items Likert-scale, the current perceived stress level. A sum score is calculated for the scale, where the maximum total value is 16. We transformed the scale to a dichotomized variable. Thus, the upper quartile is assumed to be a load factor of high-perceived stress (total score ≥ 12). *Traumatic experiences during childhood* are assessed through two binary questions concerning physical or sexual abuse during childhood and adolescence. *Stress and violent experiences within intimate partner relationships* are also assessed through four binary questions (three questions with regard to the current relationship and one with regard to IPV ever). *Substance abuse* (smoking, alcohol, drugs) is also recorded through three binary questions regarding maternal abuse and three questions regarding paternal abuse. When a question is positively answered, there is the option to specify the kind and quantity of substance but this information is not included in the analysis. *Mental health* is assessed through four binary questions (ever had a psychiatric diagnosis, ever received inpatient therapy, ever used psychotropic drugs, ever asked for psychological help). The option to specify is also given here, but again it is not included in the analysis. The questionnaire concludes with an open question concerning mother’s wishes for support during pregnancy and for the future with the baby. For an overview of the different items please see Table 1.

**Table 1:** **Overview of the risk areas, scales, number of items and the risk definition**

	**Risk Area**	**Number of Items**	**Scale**	**Definition as a risk**	**Items included in the KINDEX Sum Score**
1	Age	1	Ordinal	≤21	1
2	Migration	2	Binary	Immigration mother or father	2
3	Single parent	1	Binary	Single parent	0^1^
4	Financial problems	2	Binary	Worry about financial problems	2
			Binary	Housing index ≤ 0.5 (rooms / person)	
5	Physical symptoms, complications, medical risks	3	Binary	Physical Symptoms, complications, medical risks	3
6	Complicated prenatal bonding	5	Binary	Unplanned Pregnancy	5
			Ordinal	Concerns 7–10 (mother and father)	
				Joy 0–3 (mother and father)	
7	Current stress	4	Ordinal	PSS-4 sum score ≥ 12	1
8	Traumatic experiences during childhood	2	Binary	Physical abuse	2
				Sexual abuse	
9	Intimate partner violence (IPV)	4	Binary	Increasing number of disputes; vociferous fights in the past 8 weeks; fights including physical violence in the last 8 weeks; physical violence in a past relationship.	4
10	Substance Abuse	6	Binary	Nicotine, alcohol, drugs/mother and father.	5^2^
11	Mental Illness	4	Binary	Ever-psychiatric diagnosis, inpatient treatment, psychotropic drugs, asked for help (psychotherapy or counseling center).	3^3^

Sample descriptives and differences in risk reports between group who participated only in the KINDEX interview and the group who participated in both the Kindex and Validation Interview.

Note: ^1^ the item is excluded from the reliability analysis, all the women lived with their partners, ^2^ the item for mothers’ drug use is excluded from the reliability analysis, none of the participants was consuming illicit drugs, ^3^the item for inpatient treatment is excluded none of the participants was ever inpatient in a psychiatric clinic.

Calculating Cronbach’s alpha was achieved after recoding the ordinal scales into binary as described above. Three variables were excluded from the reliability analysis because they had zero variation; the “single parent,” (all the women lived with their partner), the “illegal drug consumption” and the “previous psychiatric hospitalization” (none of the participants were using illega drugs or had ever received psychiatric inpatient treatment). The analysis therefore consisted of 28 variables (see Table 1). The Cronbach’s coefficient value was *α = .67* for the 28 items in the KINDEX.

### Validation interview

The validation interview consisted of different standardized instruments and half-standardized tools. Sociodemographic information was collected through half-standardized questions created to assess age, education level and working situation of parents, marital state, previous and current pregnancy as well as self-reported health condition of the participant.

The standardized questionnaires used are briefly described below.

*Stress* was assessed through the Perceived Stress Scale (PSS-14) [56]. The items are related to the last month. The 14-item version has good validity and test-retest reliability (*r* = .85), and internal consistency of *α* = .84. PSS-14 scores are obtained by reversing the scores on the seven positive items and then summing across all 14 items. Possible scores range from 0–56. The PSS-14 demonstrated high internal consistency in (Cronbach’s α = .76) our study’s sample.

In addition to the PSS-14, the Everyday Stressors Index (ESI), [57] was used. The ESI consists of 20 items on a 4-point scale ranging from 0 (not bothered at all) to 3 (bothered a great deal). A composite score of everyday stressors is derived by summing responses to all items. Possible scores range from 0–60. As the ESI was originally created in English, in this study we used a validated version in Spanish, provided by the author who conducted the adaptation into Spanish in a previous study (C. Hopenhayn, Unpublished thesis). The ESI demonstrated high internal consistency (Cronbach’s *α* = .85) for the sample of our study.

The “*global stress*” value was created by summing up the z-transformed sum score of the PSS-14 and the z-transformed sum score of the ESI.

To assess *childhood abuse and neglect* we used the Checklist of Family Violence, an instrument used in previous studies in different countries and cultures [58,59]. The questionnaire consists of five subscales that assess *physical abuse, verbal-emotional abuse, sexual abuse, witnessed violence* and *neglect during childhood*. The scores for each scale are obtained by summing across items and then all the scales’ scores were summed up to calculate the overall sumscore of the CFV. The CFV demonstrated high internal consistency (Cronbach’s α = .86) in our study’s sample.

*Traumatic events and Posttraumatic Stress Symptoms* were assessed by the Posttraumatic Stress Diagnostic Scale (PDS) [60]. The instrument consists of four sections. Part 1 is a trauma checklist consisting of 12 items. In Part 2, DSM-IV criterion A2 is explored. Part 3 consists of 17 items rating the severity of DSM-IV PTSD symptom from 0 (“not at all or only one time”) to 3 (“5 or more times a week / almost always”). Part 4 assesses interference of the symptoms with all day functioning. The PDS yields a total symptom severity score (ranging from 0 to 51) that reflects the frequency of the 17 symptoms of PTSD according to DSM-IV [61]. In this study we used the Spanish Version of the PDS previously used in a study with the Mexican Population [62]. The PDS symptom score demonstrated high internal consistency (Cronbach’s *α* = .82) for our study’s sample. The “*global trauma load*” value was created by summing up the z-transformed sum score of traumatic experiences according to the PDS event-list and the z-transformed sum score of the CFV (experiences of family violence).

Various instruments were used in addition to assess *psychopathology symptoms*. For the assessment of anxiety and depression, the Spanish version of the Hopkins Symptom Checklist 25 (HSCL-25) was used [63]. It consists of 25 items: Part I of the HSCL-25 has 10 items for anxiety symptoms; Part II has 15 items for symptoms of depression. All items can be rated on a Likert-scale ranging from 1 (“Not at all”) to 4 (“Extremely”). By summing up the items a score for anxiety ranging from 10 to 40 and a score for depression ranging from 15 to 60 can be calculated. The validity of the instrument is well established and there is evidence for good test-retest reliability for anxiety (*r* = .75) and depression (*r* = .81). Both scales demonstrated high internal consistency (Cronbach’s *α* = .85 for anxiety and *α* = .80 for depression) for our study’s sample.

To assess *somatization* symptoms we used the somatization subscale of the Spanish Version of the SCL-90-R [64] which consists of 12 items rated on a 5-point scale, ranging from 0 = not at all, to 4 = extremely. The score is calculated by summing across the 12 items, possible scores can range from 0–48. Previous studies have demonstrated the reliability and validity of the SCL-90-R [64]. The somatization scale of the SCL-90-R demonstrated high internal consistency (Cronbach’s α =. 82) for our study’s sample.

The *global psychopathology* value was calculated by summing up the z-transformed sum score of the somatization subscale of the SCL-90, the z-transformed sum score of the HSCL-25 (depression and anxiety) and the z-transformed sum score of the PDS-symptoms (posttraumatic symptoms).

### Sample

One hundred nineteen pregnant women with an average age of 32 years (range: 20–42, *SD* = 4.95) and average gestational age of 31 weeks (range: 24–36, *SD* = 2.05) that attended the Maternity Hospital in Granada were interviewed using the KINDEX Spanish Version. Detailed sample description as collected from the KINDEX is presented in Table 2.

**Table 2:** **Overview of the risk factors in the KINDEX**

**Load Factors**	**Item**		**KINDEX Mum Screen**	**Val Yes**	**Val No**	**StatisticsGroup differences**
Gestational Age	In months	M (SD)	31,10 (2,06)	30,78 (2,29)	31,53 (1,63)	ns
Alter	Age in Years	M (SD)	31,86 (4,96)	31,97 (4,68)	31,71 (5,33)	ns
Migration	Mother	N (%)	5 (4,2%)	2 (3,0%)	3 (5,8%)	ns
	Father	N (%)	7 (5,9%)	5 (7,5%)	2 (3,8%)	ns
Single Parent	Not living with the father	N (%)	0 (0%)	0 (0%)	0 (0%)	ns
Financial Worries	Housing index ≤ 0,5 (Room / Person)	N(%)	7 (5,9%)	5 (7,5%)	2 (3,8%)	ns
	Financial Worries	N(%)	8 (6,7%)	5 (7,5%)	3 (5,8%)	ns
Physical Complaints and Medical risk factors	Physical Complaints	N (%)	65 (54,6%)	36 (53,7%)	29 (55,8%)	ns
	Complications	N (%)	47 (39,5%)	26 (38,8%)	21 (40,4%)	ns
	Medical Risk Factors	N (%)	32 (26,9%)	20 (29,9%)	12 (23,1%)	ns
Prenatal Bonding	Unplanned Pregnancy	N (%)	19 (16%)	13 (19,4%)	6 (11,5%)	ns
	Joy Mother (0 to 10)	M (SD)	7,66 (2,32)	7,60 (2,32)	7,75 (2,34)	ns
	Worries Mother (0 to 10)	M (SD)	6,02 (2,71)	6,03 (2,54)	6,00 (2,94)	ns
	Joy Father (0 to 10)	M (SD)	9,18 (1,52)	9,09 (1,71)	9,31 (1,25)	ns
	Worries Father (0 to 10)	M (SD)	5,28 (3,09)	5,55 (3,11)	4,92 (3,07)	ns
Stress	PSS-4 Sum Score	M (SD)	3,72 (2,62)	3,69 (2,90)	3,77 (2,24)	ns
Abuse in Childhood	Physical Maltreatment	N (%)	14 (11,8%)	10 (14,9%)	4 (7,7%)	ns
	Sexual Abuse	N (%)	2 (1,7%)	0 (0%)	2 (3,8%)	ns
Intimate Partner Conflict and Violence	Increase in Conflicts (past 8 weeks)	N (%)	18 (15,1%)	9 (13,4%)	9 (17,3%)	ns
	Vociferous Conflicts (past 8 weeks)	N (%)	13 (10,9%)	8 (11,9%)	5 (9,6%)	ns
	Physical Violent Conflict (past 8 weeks)	N (%)	1 (0,8%)	0 (0%)	1 (1,9%)	ns
	Ever violent intimate partner relationship	N (%)	6 (5,0%)	2 (3,0%)	4 (7,7%)	ns
Nicotine, Alcohol and Drugs	Smoking (pregnant)	N (%)	3,72 (2,62)	8 (11,9%)	11 (21,2%)	ns
	Alcohol (pregnant)	N (%)	14 (11,8%)	1 (1,5%)	1 (1,9%)	ns
	Smoking (father)	N (%)	2 (1,7%)	18 (26,9%)	16 (30,8%)	ns
	Alcohol (father)	N (%)	18 (15,1%)	7 (10,4%)	1 (1,9%)	ns
	Drug consumption (father)	N (%)	13 (10,9%)	3 (4,5%)	2 (3,8%)	ns
Psychiatric History	Ever psychiatric Diagnosis	N (%)	29 (24,4%)	18 (26,9%)	11 (21,2%)	ns
	Ever Psychotropic medicine	N (%)	21 (17,6%)	13 (19,4%)	8 (15,4%)	ns
	Ever inpatient psychiatric treatment	N (%)	0 (0%)	0 (0%)	0 (0%)	ns
	Ever sought psychological help	N (%)	19 (16%)	11 (16,4%)	8 (15,4%)	ns
KINDEX	KINDEX Sum Score	M (SD)	4,19 (2,75)	4,24 (2,82)	4,13 (2,67)	ns

Sample descriptives and differences in risk reports between group who participated only in the KINDEX interview and the group who participated in both the Kindex and Validation Interview and the validation interview and the group who only participated in the KINDEX interview.

Note: *M* (Mean), *SD* (Standard Deviation), *N %* (Number of Participants in percentages), *Val Yes* (values for participants in the validation interview), *Val No* (values for participants only in the Kindex interview) *ns* (not significant).

### Statistical analysis

Statistical analysis was performed using SPSS 21st Version.

Sum scores of the standardized instruments used in the validation interview were *z*-transformed and *z* values were summed up to create three global values.

Afterwards we explored the normality assumption through the Kolmogorov-Smirnov normality test for the global stress, psychopathology and trauma load values as well as for the KINDEX sum score. The K-S test values were: for the global stress *D*(50) = .13; *p* = .005 for the global psychopathology *D*(66) = .16; *p* ≤ .001, for the global trauma load *D*(66) = .16; *p* ≤ .001 and for the KINDEX sum score *D*(66) = .16; *p* = .005. Significant values indicate that the normality assumption was not met. Consequently we only used non-parametric testing for group comparisons (Mann-Whitney-U and Kruskal-Wallis H) and correlations (Spearman’s rank (rho) correlation coefficient).

To examine the frequency of risk factors reported by our sample in the KINDEX interview we performed descriptive statistics. For the comparison of the group of women that only took part in the KINDEX interview and the group of women that took part in both the KINDEX interview and the validation interview (see Table 2) we conducted Chi-Square Tests for dichotomous variables and Mann-Whitney-U tests for linear variables.

To examine the concurrent validity of the KINDEX, a sum score was calculated including the 31 dichotomous items (see Table 1), (*M* = 4.24, min = 0, max = 14, SD = 2.82) for the group participating in the validation interview. The sum score was then correlated with the global stress score, the global trauma load score and the global psychopathology score as assessed in the validation interview. The global score of the validation interview are presented in Table 3.

**Table 3:** **Means, (±SD) of the sample in the variables assessed in the validation interview**

**Scale**	**N**	**M**	**SD**	**Mdn**	**Min**	**Max**
PSS-14 (Stress)	67	25.88	4.71	19.0	1	36.0
ESI (Stress)	67	29.14	7.13	26.0	20	57.0
Global stress	67	.00	1.74	-.35	-3.30	6.11
HSCL-Depression	67	6.20	5.62	5.0	0	24.0
HSCL-Anxiety	67	4.04	4.68	3.0	0	19.0
SCL-Somatization	67	10.62	8.56	8.0	0	37.0
PDS-PTSD symptoms	66	2.13	3.74	.00	0	18.0
Global psychopathology	66	-.06	3.15	-.96	-3.42	9.49
CFV (Child Maltreatment)	67	2.83	3.46	1.00	0	15.0
PDS (Traumatic Events)	67	1.92	1.52	2.00	0	5.0
Global trauma load	67	.001	1.70	-.48	-2.08	4.87

Note: *N* (number of participants), *M* (mean), *SD* (standard deviation), *Mdn* (Median), *Min* (score minimum), *Max* (score maximum), *PSS-14* (perceived stress scale-14 items), *ESI* (everyday stress index), *HSCL* (hopkins symptoms checklist), *SCL* (symptom checklist), *PDS* (posttraumatic stress diagnostic scale), *CFV* (checklist of family violence).

In addition, we examined if participants who reported having two of the most important risk factors in the KINDEX also have higher means in the respective validation scales. The items we chose to include in this analysis were: “ever received a psychiatric diagnosis”, “ever have experienced physical violence during childhood”. We expected participants who report a previous psychiatric diagnosis (as assessed in the KINDEX) to present higher scores of somatization (Subscale of the SCL-90; Symptom Checklist), posttraumatic stress symptoms (PDS; Posttraumatic Diagnostic Scale), anxiety and depression (Anxiety and Depression subscales; HSCL-25) in the validation interview. In the same way participants who report in the KINDEX having experienced physical abuse in childhood, they were also expected to have higher scores in the related subscale of the Checklist of Family Violence (CFV). To examine this assumption we used the Mann-Whitney-U test.

Kruskal-Wallis H test between subjects was conducted to compare the effect of the hospital-unit where the interview was carried out on the KINDEX sum score.

Only one missing value for one participant was found in our data set, in the scale of PTSD-Symptoms, applied in the Validation Interview. We consider that this value is missing completely at random (MCAR). We address the missing data using the method of complete-case analysis.

## Results

### Concurrent validity: correlations between the KINDEX sum score and the global scores in the validation interview

**Figure 1:**
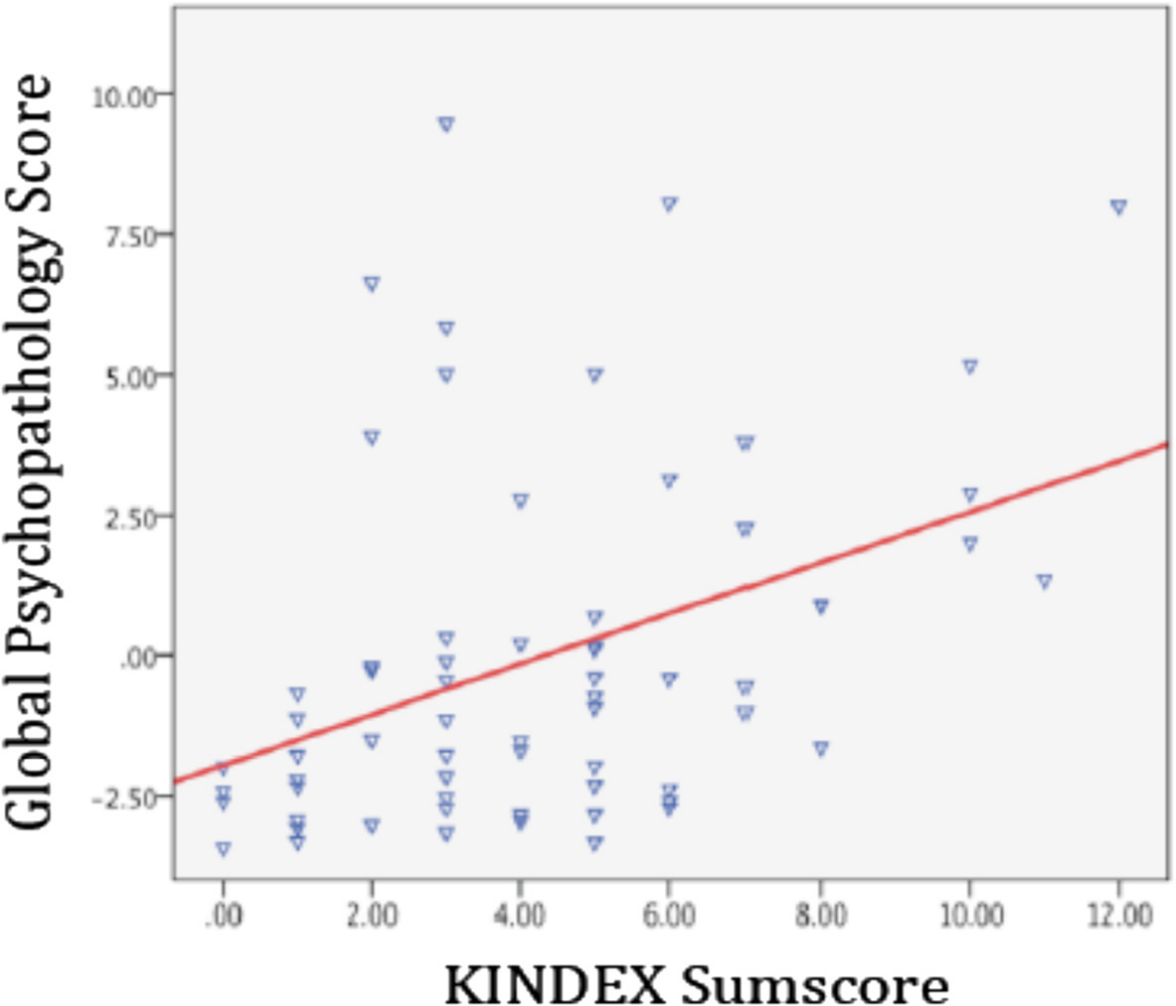
**Relation between the KINDEX sum score on the X-axis and the global psychopathology score (left Y-axis).**

**Figure 2:**
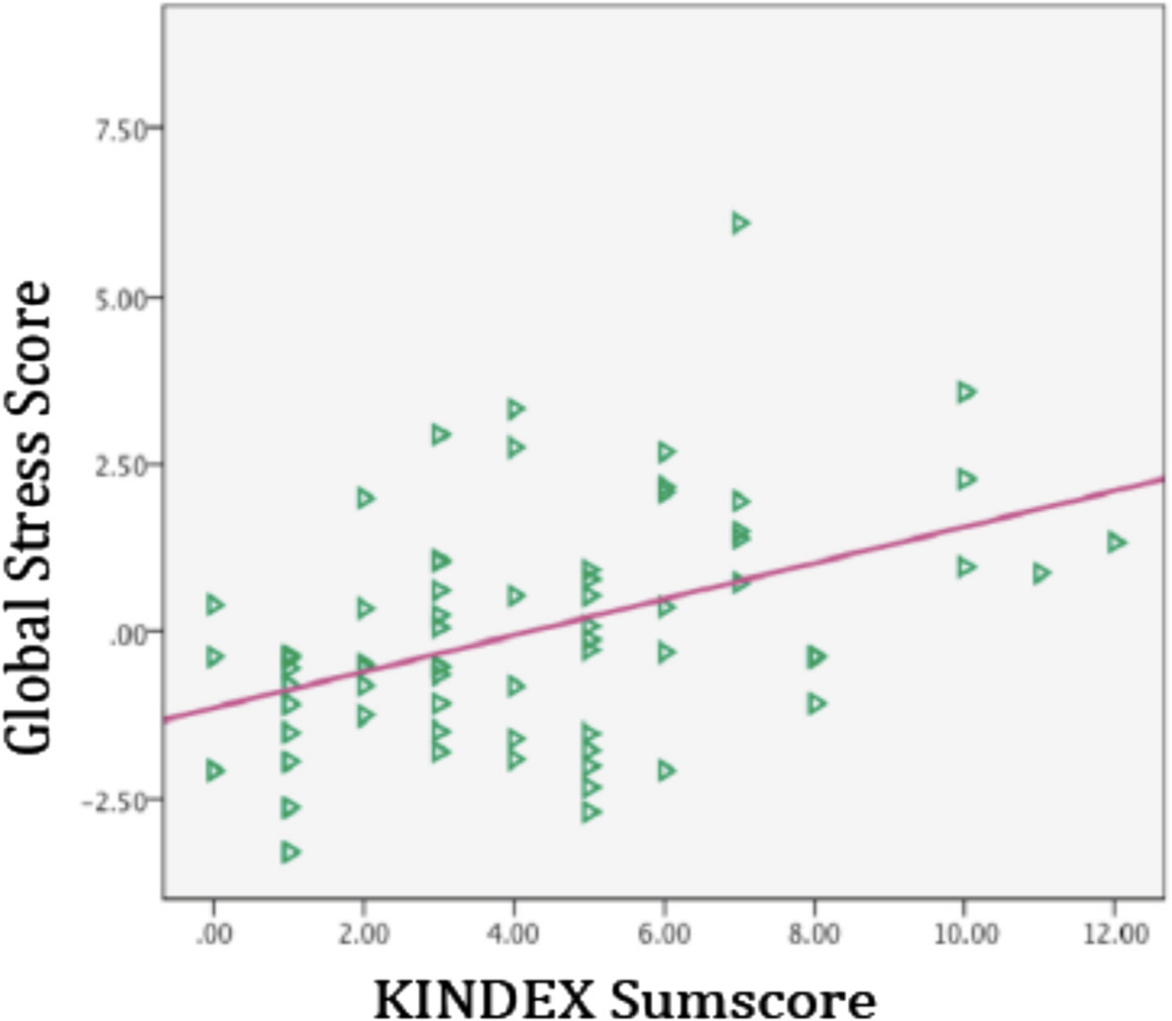
**Relation between the KINDEX sum score on the X-axis and the global stress score (left Y-axis).**

The KINDEX sum score positively correlated with the global stress score (*r* = .45; *p* ≤ .001), the global traumaload score (*r* = .38; *p* ≤ .001) and the global psychopathology score (*r* = .44; *p* ≤ .001) (see Table 4 and Figures 1 and 2).

**Table 4:** **Correlates between the KINDEX and the global stress, global psychopathology, and the global trauma load in the validation interview**

	**KINDEX Sum Score**	**Validation Global Stress Score**	**Validation Global Psychopathology Score**	**Validation Global Trauma Load**
	**N = 67**	**N = 67**	**N = 66**	**N = 67**
KINDEX Sum Score	1	.45**	.44**	.38**
Validation Global Stress Score		1	.62**	.27*
Validation Global Psychopathology Score			1	.45**
Validation Global Trauma Load				1

Note: **Correlation significant in the level of ≤ .001, bolds indicate significant correlations.

### KINDEX items’ association with the corresponding validation scales

In relation to the items referring to mental health history, as illustrated in Table 5, results indicate that there are statistically significant differences between women who have ever received psychiatric diagnosis (n = 18) and women that had not (n = 49) in the scales of somatization (*U* = 255.5; *p* = .009) PTSD symptoms (*U* = 164.0; *p* ≤ .001) and depression (*U* = 284.0; *p* = .02) but no statistically significant differences were observed in the anxiety scale (*U* = 325.0; *p* = .09).

**Table 5:** **Group Comparisons between women with and without psychiatric diagnosis in the past and different mental health scales in the validation interview**

	**KINDEX: Psychiatric diagnosis ever**							
	**YES N = 18**			**NO N = 49**				
**Validation Instrument**	**Mdn**	**Min**	**Max**	**Mdn**	**Min**	**Max**	**U**	**p**
**SCL-Somatization**	**11**	**3**	**37**	**7**	**0**	**12**	**255.0**	**.009**
**PDS- PTSD Symptoms**	**2**	**0**	**18**	**0**	**0**	**12**	**164.0**	**≤.001**
**HSCL-Depression**	**7**	**0**	**19**	**4**	**0**	**24**	**284.0**	**.02**
HSCL-Anxiety	4	0	19	2	0	18	325.0	.09

Note: *N* (number of participants), *Mdn* (median), *Min* (score minimum), *Max* (score maximum), *U* (Mann-Whitnery U value) *p* (level of significance), *HSCL* (Hopkins symptoms checklist), *SCL* (symptom checklist), *PDS* (posttraumatic stress diagnostic scale), *YES* (participants answered item positively) *NO* (participants answered item negatively), bolds indicate significant differences.

Regarding the item related to physical violence in childhood, as illustrated in Table 6, results indicate that there are statistically significant differences between women who reported having experiences of physical violence in childhood (n = 10) and those that did not (n = 57) in relation to the sum score of the CFV (*U* = 96.0; *p* ≤ .001), the subscales of *physical violence* (*U* = 92.0; *p* ≤ .001), *witnessed violence* (*U* = 84.5; *p* ≤ .001) and *verbal emotional violence* (*U* = 107.5; *p* ≤ .001). No significant differences were found between the two groups in relation to the subscales of neglect (*U* = 265.5; *p* = .33) and sexual abuse (*U* = 280.0; *p* = .67) – see also Tables 5 and 6.

**Table 6:** **Group comparisons between the group reporting childhood physical violence and the group reporting none, in relation to the corresponding scales in the validation interview**

	**KINDEX: Physical violence in childhood**							
	**YES N = 10**			**NO N = 57**				
**Val. Instrument**	**Mdn**	**Min**	**Max**	**Mdn**	**Min**	**Max**	**U**	**p**
**CFV- Physical Violence**	**4.0**	**0**	**7**	**0**	**0**	**9**	**92.0**	**<.001**
**CFV- Witnessed Violence**	**1.5**	**0**	**3**	**0**	**0**	**2**	**84.5**	**<.001**
**CFV- Verbal Emotional Violence**	**3.0**	**0**	**4**	**1**	**0**	**3**	**107.5**	**≤.001**
CFV- Neglect	0	0	2	0	0	1	265.5	.33
CFV – Sexual Abuse	0	0	2	0	0	0	280.0	.67

Note: *n* (number of participants), *Mdn* (median), *Min* (score minimum), *Max* (score maximum), *U* (Mann-Whitnery U value) *p* (level of significance), *CFV* (checklist of family violence), *YES* (participants answered positively in item) *NO* (participants answered negatively in item), bolds indicate significant differences.

## Discussion

Although there is sufficient and convincing scientific evidence that prenatal risk factors can have a lifelong adverse impact on the unborn, this information is still not routinely collected within antenatal health care. Although some outcomes indicated the efficacy of the use of specific screening tools and prevention programs [51], to our knowledge, there is no other short instrument than the KINDEX that has been applied in European countries and is able to identify risks from eleven psychosocial areas known to threaten the healthy development of individuals over their life span. Most of the assessment tools developed so far have focused on risk factors for the maternal mental health in the postpartum period.

In our study, we present the cultural adaptation of the KINDEX to the Spanish public health setting. This is a new prenatal assessment tool for psychosocial risk factors for both the maternal mental health and the child development in the long run. This tool was originally developed and validated in Germany [65] and has been designed as a short interview (20–30 min) that can be conducted by midwives and gynecologists without any specific training in psychosocial concepts. The medical staff included in our study reported experiencing no problems in carrying out the KINDEX interviews throughout the project and continued the interviews assigned to them until the conclusion of the study. Even though the time required for its use in the German population was 20–30 minutes, the majority of the medical collaborating in the Spanish study stated an approximate time of 15 minutes and noted that it did not interrupt the normality of their clinical praxis. Midwives and gynecologists facilitated the interview process during outpatient consultations. The interviews carried out with hospitalized pregnant women were demonstrated feasible since midwives could arrange the interview at a more “relaxed” time during their shift. Midwives who interviewed women undergoing special medical screening (eg. gestational diabetes, high blood pressure) in the fetal medicine unit did not report any problems with the time spent administering the KINDEX. None of the interviewers dropped-out from the project, which indicates acceptance of the KINDEX tool by midwives and gynecologists in the public health setting. The high feasibility and acceptance of the KINDEX is relevant for its application in the hospital setting. The structure of the hospital and the involvement of the four units in the interviews bolster our conclusion that the KINDEX can be embedded in public health centres successfully. Likewise, the involvement of pregnant women in the interview was very satisfactory, since no dropouts were registered once the women joined the study. Based on this, we conclude that the implementation of the KINDEX, as a prenatal screening tool in the Spanish public health sector is quite feasible. We recommend further research in a variety of health contexts regarding the feasibility and acceptance of the application of the Kindex, especially for General Practitioners of primary care, who often have first contact with the women.

Many studies have supported the fact that prenatal screening for and management of depression and anxiety are very important to prevent adverse maternal mental health [66,47,48] and psychosocial screenings to identify women at risk [67,10]. Determining the level of risk (measured as number of risks) triggering the initiation of referral pathways to the corresponding mental and social services of each health centre is a challenging task. Psychosocial assessments leading to the referral of women in high risk involve several health sectors. The delivery of appropriate interventions requires proactive collaboration of a multidisciplinary group of professionals. Nevertheless the activation of this referral system and intervention with women in risk is beyond the aims of this study, while this was examined in the validation of the KINDEX in Greek, developed in public health centres in Crete Island [68]. In the Greek study, medical staff was encouraged, based on the KINDEX assessment, to refer pregnant women that presented 2 or more risks. Results showed that the medical staff correctly identified women at risk, and referred them to mental health services, though these women did not follow through. Because of this, we believe that a successful assessment, referral and intervention program can provide only the frame of general perinatal clinical guidelines. In Australia such guidelines have been recently been established for the treatment of perinatal mental health conditions [69], these are yet not established in Spain and in many other European countries.

To assess the validity of the data collected with the KINDEX a randomized subsample of pregnant women was additionally interviewed by a trained clinical psychologist using different standardized instruments to assess three major risk areas, namely stress, psychopathology and trauma load. Moderately high, positive correlations between the KINDEX sum score and the global stress, global psychopathology and global trauma load assessed in the validation interview, indicate that the KINDEX has good concurrent validity. In addition, exploratory analysis of single items in our study showed that women who reported a history of a psychiatric diagnosis (KINDEX assessment) report current higher levels of somatization, PTSD symptoms and higher levels of depression in the validation interview and as expected, participants who reported childhood physical maltreatment in the KINDEX presented higher scores in the Checklist of Family Violence subscales of *physical violence*, *witnessed violence* and *verbal emotional violence* in the validation interview. In summary, these results corroborate the validity of the data collected by medical staff when using the KINDEX instrument in a public health setting.

In addition to our statistically determined results, participants frequently reported in validation interviews with the clinical psychologist that even though the KINDEX interview conducted by midwives and gynecologists came as unexpected health service to them, they felt more cared for by the medical staff. Many also stated such enquiries made the medical treatment experience more holistic and patient-centered. This not only underlines the feasibility of the KINDEX in the public health setting but also shows that pregnant women are open to discussing their problems with medical experts and hope for support.

## Conclusions

The present study contains numerous clinical implications. Results of the validation interviews indicate that women who have suffered adverse experiences in the past (global trauma load) still show higher levels of current stress and psychopathology. It is therefore evident that pregnant women who are at risk due to past and current adverse experiences should receive adequate interventions in order to prevent further mental health problems and unfavorable development of their offspring. The KINDEX therefore can serve as a module to identify women in need not only concerning mental health problems but other social disadvantages and allow for the establishment of early support services that meet psychosocial risk situations during gestation and after birth. Appropriate cost-effective interventions during this early stage would mean a revolution in preventive medicine as well as create a significant impact in primary care, creating a more integrative comprehensive health attention towards pregnant women, neonates, and the family.

### Study limitations

A potential limitation of the study may be the lack of representativeness of the target group, since not all the prevalence rates revealed by the KINDEX interview are comparable with prevalence rates in the general Spanish population. For example lifetime mental disorders (24.4%) in the present study, and 21–25% in the general population [70] and childhood maltreatment (11.8% in the present study, 6.3% in the general population [71] are comparable. But the report of childhood sexual abuse (1.7% in the present study, 12–17% in the general population [72,73] and IPV (5.0% in the present study, 10.6% in the general population are lower [74]. We do not know, whether our study population was a privileged sample of a city population, or whether the lower reports of sexual abuse and IPV have to be explained by the fact that answering such questions can be upsetting for the respondents [75] and more difficult for women when interviewed face to face [76,77]. Even though the medical staff was informed about the sensitive nature of such questions and the importance of honest answers for the delivery and wellbeing of the mothers, we are not certain how these items were asked since the interview was conducted by lay-staff untrained as interviewers.

The KINDEX was used as a screening instrument for 11 risk factors in a very short length in order to be brief and easy to use. Amongst its aims is not the symptoms severity of any type of psychopathology, but the identification of the possible existence of this. Therefore it cannot be used as a clinical diagnostic tool, but as a referral tool that medical staff can use to identify such risks and refer patients to the appropriate mental and social health services for a thorough diagnostic assessment.

In this study we have not tested the sensitivity and specificity of the KINDEX which would enhance an insight into the psychometric properties of this assessment tool. Through studies using a prospective design the predictive validity, the sensitivity and specificity of the tool could be assessed.

Additional studies in other hospitals in both cities and rural Spanish settings are needed to build up a more extensive and solid database for further generalizations. In addition we also recommend longitudinal prospective studies to examine the predictive validity of the KINDEX on the child’s development and the mother-child relationship.

Despite these limitations, this is the first study that shows the feasibility and validity of a prenatal assessment tool for psychosocial risks in Spain in a general public health setting. The risk factors assessed by the KINDEX are based on a systematic review of the empirical literature and the fact that these risk factors can be validly assessed by non-trained midwives and gynecologists in a short standardized interview of only 15–30 minutes is an encouraging result and builds the first step in revolutionizing the primary care for pregnant women and their offspring’s outcomes.

## Endnotes

^a^ On Monday the first pregnant woman, on Tuesday the second, etc.
